# Dietary Patterns in Acne and Rosacea Patients—A Controlled Study and Comprehensive Analysis

**DOI:** 10.3390/nu15204405

**Published:** 2023-10-17

**Authors:** Anne Guertler, Arina Volsky, Quirine Eijkenboom, Tobias Fiedler, Lars E. French, Markus Reinholz

**Affiliations:** 1 Department of Dermatology and Allergy, LMU University Hospital, LMU Munich, 80539 Munich, Germany; 2 Dr. Phillip Frost Department of Dermatology and Cutaneous Surgery, Miller School of Medicine, University of Miami, Miami, FL 33136, USA

**Keywords:** acne, rosacea, diet, nutrition, clinical score, inflammatory dermatoses

## Abstract

As the relationship between exposome factors and inflammatory skin diseases is gaining increasing attention, the objective of this study was to investigate dietary patterns among acne and rosacea patients and to establish the disease risk attributable to nutrition. In this cross-sectional, controlled study, patients’ dietary habits were assessed via subjective ratings of beneficial and trigger foods, followed by standardized food frequency surveys (FFS). Scores for disease-specific risk stratification based on dietary habits were proposed. Clinical assessments, dermatologic examinations, and laboratory analyses were performed. A total of 296 patients (acne group (AG) n = 120, control group (ACG) n = 32; rosacea group (RG) n = 105, control group (RCG) n = 39) were included. The significant impact of diet on disease severity was self-reported by 80.8% of the AG and 70.5% of the RG. Leading dietary triggers were found in both groups, while beneficial food items were identified more clearly by the AG. FFS revealed significant dietary differences between the AG, RG, and control groups. Disease-specific scores showed greater precision for acne (odds ratio 14.5 AG, 5.5 RG). The AG had higher insulin-like growth factor (IGF)-1 levels correlating with dairy intake (*p* = 0.006). Overall, this study underlines the influence of diet on acne and rosacea, providing valuable disease-specific scores for dietary risk stratification. Consuming vegetables, legumes, oily fish, olive oil, and nuts, and limiting meat, cheese, and alcohol appear to be beneficial for both acne and rosacea. Future studies can build on these data to further improve preventive and therapeutic strategies.

## 1. Introduction

Given the growing importance of exposome factors, including nutrition, in disease prevention and treatment, there is an increasing demand to explore its relationship with the most common skin conditions such as acne vulgaris and rosacea [1,2]. These facial dermatoses continue to gain attention as patients seek guidance from dermatologists on optimal nutrition to complement their treatment regimens [3]. However, assessing the impact of diet on disease severity remains challenging due to the subjective nature of self-reported data, insufficient contemporary data on patients’ dietary habits, and the lack of standardized disease-specific nutritional protocols. Large observational controlled studies are therefore necessary to report associations between certain foods and disease risk, allowing valuable insights for preventive and therapeutic strategies to complement prescription medication [4].

While the relationship between diet and inflammatory facial skin conditions is not fully understood, recent studies have demonstrated that certain dietary factors may influence their development and severity [5]. For acne, consumption of highly processed carbohydrates, milk, and dairy products have been identified as dietary triggers [6]. Their intake should be limited because of their role in increasing insulin, a hormone produced by the pancreas that is important for regulating blood glucose levels, and insulin-like growth factor (IGF)-1, a growth factor produced primarily by the liver and involved in multiple anabolic pathways. Fluctuations in insulin and IGF-1 levels can affect seborrhea, one primary cause of acne [7,8]. In addition, these fluctuations can stimulate the release of pro-inflammatory molecules, which are known to contribute to the development of acne and rosacea [9]. While studies have reported a positive correlation between plasma IGF-1 levels and the clinical severity of acne [10], insulin resistance has recently been reported in rosacea patients [9,11]. Overall, the understanding of the impact of diet on rosacea remains limited compared to acne and despite ongoing research efforts. Capsaicin-, heat-, or alcohol-related nutritional triggers may be intertwined with the multifactorial pathophysiology of rosacea, including dysbiosis of the skin microbiome, vascular malformation, and immune dysregulation, yet their impact varies greatly between individuals [12,13]. Overall, comprehensive dietary recommendations for rosacea are still deficient [14,15].

The present study aims to provide a comprehensive analysis of the dietary habits of patients with acne and rosacea. Its objective is to establish more precise dietary recommendations specifically tailored for each facial dermatosis and propose standardized dietary scores that clinicians can utilize to evaluate the risk for acne and rosacea attributable to patients’ dietary habits. By adopting this systematic approach, personalized treatment strategies can be devised, ultimately resulting in enhanced patient outcomes.

## 2. Materials and Methods

### 2.1. Primary Objective

The primary objective was to investigate dietary patterns in acne and rosacea patients in comparison to healthy control groups to improve dietary recommendations.

### 2.2. Secondary Objectives

Secondary objectives were defined as follows:

- To introduce clinical scores determining the risks for acne and rosacea based on patients’ dietary habits;
- To rate participants’ quality of life;
- To evaluate the clinical severity of participants’ skin conditions;
- To compare patients’ facial sebum values and laboratory parameters.

### 2.3. Study Design

This cross-sectional, single-center, exploratory, controlled study was conducted at the Department of Dermatology and Allergy of the Ludwig-Maximilians-Universität (LMU), Munich, Germany, between September 2020 and June 2021. The study included four groups: the acne group (AG) and the rosacea group (RG), consisting of individuals aged 12 years and older, regardless of their current clinical severity and ongoing treatment. Control groups were established for each AG (ACG) and RG (RCG) by recruiting healthy participants without a history of facial dermatoses, matched to the AG and RG in terms of gender, age, and body mass index (BMI). Exclusion criteria for all groups were pregnancy and breastfeeding. The study adhered to the principles of the Declaration of Helsinki and received ethical approval from the Ethics Committee of the Faculty of Medicine (Ref.-No. 20-585). Written informed consent was obtained from all participants and, in the case of minors, from their legal guardian prior to their inclusion in the study.

### 2.4. Assessments and Outcomes

#### 2.4.1. Dietary Assessment

Participants in the AG and RG were asked to subjectively rank different food items using a detailed questionnaire based on whether they had a positive or negative effect on their respective clinical severity. Alcohol triggers were broken down into more detail in the RG. Moreover, the dietary habits of all participants (AG, RG, and control groups) over the last four weeks were assessed using a standardized food frequency survey (FFS) comprising 125 detailed questions. The FFS provided insights into the overall intake and frequency of various foods and beverages [16,17]. Based on the FFS data, each patient received personalized nutritional counselling from a doctor specialized in clinical nutrition to improve their eating habits according to individual needs.

#### 2.4.2. Nutrition Scores

Clinical nutrition scores were developed based on patients’ dietary habits as a predictive tool for assessing the risk for acne and rosacea: the acne nutrition score (ANS) and the rosacea nutrition score (RNS). The development process involved employing the Receiver Operating Characteristic (ROC) analysis for each food item [18]. Data collected from the FFS were utilized to identify significant variables that differed between the AG and ACG, as well as between the RG and RCG. The resulting Area Under the Curve (AUC) values were evaluated to determine the accuracy and predictive ability of the clinical scores. Only significant variables (*p* < 0.05 for acne and *p* < 0.1 for rosacea) with an AUC value greater than 0.6 were selected as contributors to the respective scores. These AUC values served as a measure of how effectively the clinical scores could differentiate between patients with acne/rosacea and their respective control groups. Furthermore, the Youden Index was used to calculate cut-off frequencies to determine the optimal threshold for classifying patients based on the clinical scores. These cut-off frequencies enhanced the clinical utility of the scores by providing a standardized criterion for classification.

#### 2.4.3. Questionnaires

All groups were asked to complete a comprehensive study-specific questionnaire, which was developed by the investigators and utilized to gather detailed information on demographics, medical history, current, and previous treatments. The Dermatology Life Quality Index (DLQI) was employed as a standardized tool to assess the impact on patients’ quality of life, including psychological disability at work, social and sexual relationships, depression, and anxiety [19]. The DLQI scores ranged from 0 to 30, with higher scores indicating a greater impact on quality of life (0–1: no effect, 2–5: small effect, 6–10: moderate effect, 11–20: very large effect, 21–30: extremely large effect). Additionally, the EuroQol questionnaire (EQ-5D-EL) was used to evaluate the overall health status of the patients. This questionnaire consisted of five dimensions (mobility, self care, usual activities, pain/discomfort, anxiety/depression) and three corresponding levels (no problems, some problems, extreme problems) [20].

#### 2.4.4. Dermatological Severity

The predilection sites of acne (face, chest, back) and rosacea (face, chest, eyes) were examined in the AG and RG, and the overall clinical severity was classified by an independent dermatologist. For acne, the number of non-inflammatory lesions (comedones) and inflammatory lesions (papules, pustules) were counted to assess clinical severity [21]. Acne was also classified into its three main subtypes (acne comedonica, acne papulopustolosa, acne conglobata). To evaluate clinical severity in rosacea patients, the global ROSacea COnsensus panel (ROSCO) criteria were applied [21]. In addition, patients in the AG and RG were asked to self-assess their clinical severity using a 3-point scale (mild, intermediate, severe). Digital photography was employed to ensure objective documentation of the patients’ skin condition, utilizing a Nikon D5 with an AF-S Nikkor 60 mm lens.

#### 2.4.5. Facial Sebum Levels

The Sebumeter^®^ SM 815 (Courage + Khazaka, Cologne, Germany) was used to quantitatively measure patients’ facial sebum levels in all groups. The hand-held device, which exposed a 64 mm^2^ strip of tape, was placed perpendicularly and without any pressure on three predefined areas of the patients’ skin (forehead and both cheeks). The degree of tape transparency correlated with skin sebum levels (μg/cm^2^), determined by photometry [22]. Measurements were performed under standardized conditions in an air-conditioned room.

#### 2.4.6. Body Fat Measurement

The skinfold caliper, a non-invasive handheld device, was used to accurately measure the thickness of skin folds, including the underlying layer of fat at predefined anatomic regions (triceps, biceps, subscapular, suprailiac) [23]. The measurements were obtained in millimeters (mm). Body fat percentage was calculated according to Berres [24]. Patients’ body mass index was calculated using the standard formula (BMI = weight (kg)/height (m)^2^).

#### 2.4.7. Laboratory Parameters

Blood samples of all groups were obtained according to a standardized protocol. A broad laboratory fasting panel including nutritive parameters, such as IGF-1, glucose, insulin, HbA1c, fructosamine, cholesterol, triglycerides, HOMA-index ((insulin[µU/mL] × glucose [mg/dL])/405)) [25], and inflammatory markers (CRP and total leukocyte count), as well as vitamin D and zinc levels, was analyzed [26,27,28].

#### 2.4.8. Statistical Analysis

The data analysis was conducted by a professional statistician using the SPSS software version 26 (IBM, Armonk, NY, USA). The level of significance for all statistical tests was set at a *p*-value of 0.05, ensuring robust and reliable results. Descriptive statistics, including mean, median, standard deviation, minimum, and maximum values, were calculated to summarize the demographic data. Appropriate parametric and non-parametric tests were selected and applied based on the nature of the data. Multivariate analyses were conducted to explore potential relationships between variables and outcomes of interest. Graphs were created using Microsoft PowerPoint (Version 16.54).

## 3. Results

### 3.1. Demographic Data

The study population consisted of 296 patients, including 120 individuals in the AG and 105 in the RG. These groups were compared to their respective control groups, which consisted of 32 participants in the ACG and 39 in the RCG. The demographic characteristics of the participants are summarized in Table 1. All groups were comparable in terms of their interest in nutrition, enjoyment of cooking, education level, social status, and medical comorbidities. Most patients had mild to moderate symptoms. The AG was significantly younger with lower BMI and body fat percentage compared to the RG (*p* < 0.001). Acne patients rated their skin appearance as significantly worse than the independent dermatologist (*p* < 0.001), while no difference was observed in the rosacea cohort. Both the AG and RG had significantly higher facial sebum levels compared to their respective control groups (*p* = 0.002, *p* = 0.006), while there was no difference between the AG and RG (Table 1).

### 3.2. Nutrition

#### 3.2.1. Subjective Assessment

**Figure 1:**
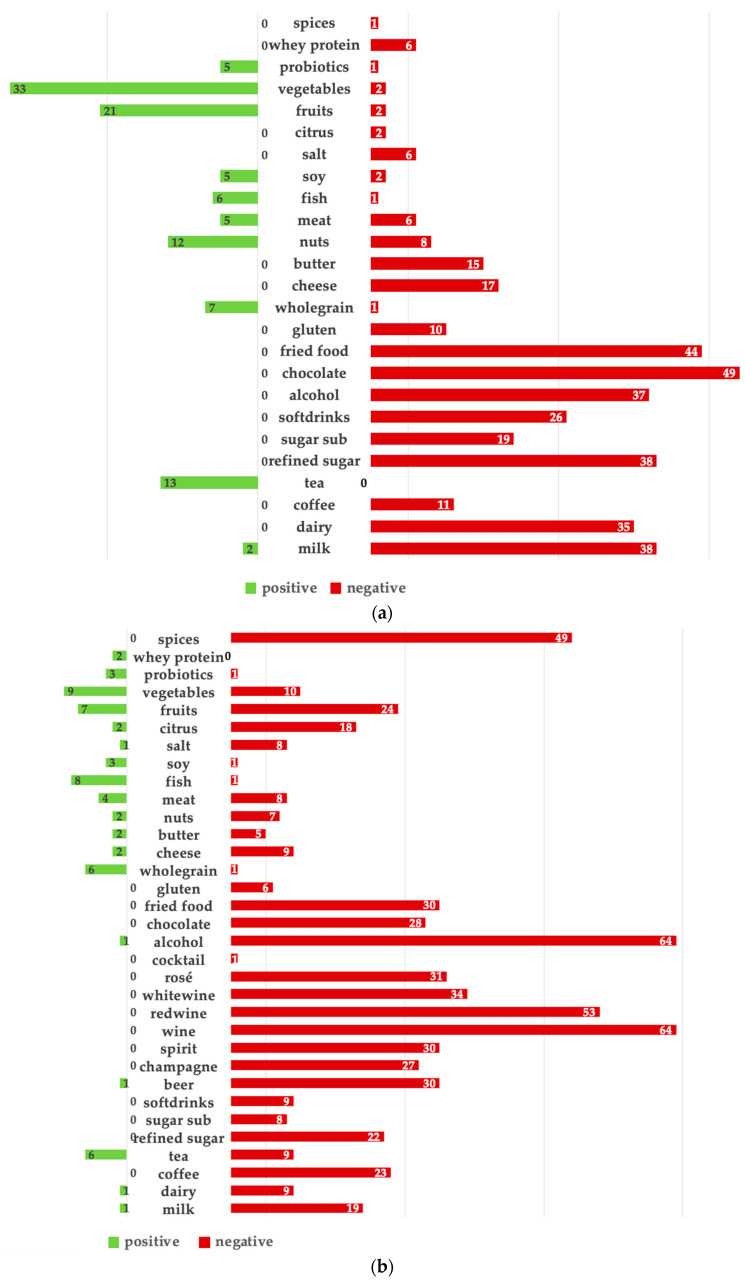
(**a**): Acne patients’ subjective assessment of food items with beneficial (green) and negative impact on their clinical severity (red). (%). (**b**): Rosacea patients’ subjective assessment of food items with beneficial (green) and negative impact on their clinical severity (red). (%).

According to 80.8% of the AG and 70.5% of the RG, diet played a role in the clinical severity of acne and rosacea (Figure 1a,b). Chocolate, fried foods, refined sugar, and milk were perceived as major acne triggers, followed by alcohol and dairy products.

For rosacea, alcohol was perceived as the main trigger, especially wine, followed by spices, fried foods, chocolate, coffee, refined sugar, and milk. Both the AG and RG perceived vegetables, nuts, whole grains, tea, and fish as the most beneficial for their disease, but more clearly in the AG. Fruits were perceived as beneficial for acne, while rosacea patients reported them as triggers, especially citrus fruits.

#### 3.2.2. Food Frequency Survey (FFS)

When comparing the dietary habits of the AG and ACG, significant differences were observed in 11 food items, as shown in Table 2. Acne patients consumed significantly less fruits, vegetables, boiled potatoes, pasta, and soy products than the ACG. They also reported lower intakes of water, coffee, wine, and milk. Further differentiation based on acne severity revealed that soft drink consumption was more common in patients with acne papulopustulosa (*p* = 0.020) compared to other forms of acne.

Significant results comparing dietary habits between the RG and RCG were found for eight food items, as shown in Table 3. Honey, ham, burgers, meat, and fried potatoes were consumed significantly more in the RG compared to the RCG. On the other hand, rosacea patients ate significantly less soy products and legumes and drank significantly less coffee than the RCG. Notably, the RG reported a significantly higher intake of oral supplements compared to the AG (*p* = 0.016), as shown in Table 1.

#### 3.2.3. Nutrition Scores

Cut-off consumption frequencies and corresponding odds ratios for each food item were calculated for the acne cohort (Table 4a,b) and rosacea cohort (Table 5a,b).

The ANS included 13 items, each with one point attributed. Thus, values of the score ranged from 0 to 13 (Table 6a). The score was applied to the acne cohort. The significant cut-off was calculated as ≤ 7 points (*p* < 0.001) for an increased acne risk with an odds ratio of 14.5 (Table 6b).

The RNS included seven items, each with one point attributed. Thus, values of the score ranged from 0 to 7 (Table 7a). The score was applied to the rosacea cohort. The significant cut-off was calculated as ≤4 points (*p* < 0.001) for an increased rosacea risk with an odds ratio of 5.5 (Table 7b).

#### 3.2.4. Laboratory Analysis

The AG had significantly higher IGF-1 levels compared to the ACG (*p* = 0.006), along with lower triglyceride levels (*p* = 0.009). Interestingly, patients with elevated IGF-1 levels were more likely to have acne on the chest than on the face (61.9% vs. 34.4%, *p* = 0.026), and reported a significantly higher consumption of dairy products such as butter (*p* = 0.024), cream cheese (*p* = 0.025), and cheese (*p* = 0.024) compared to acne patients with normal IGF-1 levels. Elevated levels of HbA1c were found more frequently in patients with acne conglobata and papulopustulosa compared to acne comedonica (*p* = 0.005). Patients with acne conglobata also exhibited significantly higher CRP levels than patients with acne papulopustulosa and comedonica (*p* < 0.001, respectively). No significant differences in glucose metabolism, cholesterol, vitamin D, or zinc levels were found between the AG and ACG.

Rosacea patients showed a significantly higher HOMA index (*p* = 0.005), lower triglyceride levels (*p* < 0.001), and lower zinc levels (*p* = 0.017) compared to the RCG. Elevated leukocyte levels (*p* = 0.001) were also observed in the rosacea group. When comparing the AG and RG, significantly higher IGF-1 levels were found in the AG (*p* < 0.001), as well as higher cholesterol, triglycerides, and LDL levels compared to the RG (*p* < 0.001, respectively) (Table 8).

### 3.3. Quality of Life and Overall Health

The impact of acne on patients’ quality of life was more pronounced compared to rosacea, as indicated by the mean DLQI scores (AG mean DLQI score of 6.5 with a moderate effect, RG mean DLQI score of 5.4 with a small effect, *p* = 0.006). However, no significant difference was observed in the EQ-5D-EL scores between the AG and RG (*p* = 0.177). Both the AG and RG reported a significant decrease in quality of life and overall health when compared to their respective control groups, as evidenced by the DLQI and EQ-5D-EL scores (*p* =< 0.001, respectively, Table 1). Stress was identified as a trigger for both acne (69.2% AG) and rosacea (75.2% RG).

## 4. Discussion

This cross-sectional, controlled study involved a large cohort of acne and rosacea patients to provide valuable insights for healthcare professionals and patients into the relationship between specific foods and the presence and severity of these conditions. The study used a three-step approach to dietary analysis. First, a subjective assessment was conducted in which patients reported the effect of various foods on the clinical severity of their disease. Second, patients’ dietary intake was assessed using a standardized FFS and compared to a control group. Finally, the FFS reports were translated into clinical scores that served as tools to predict the risk for acne and rosacea based on dietary habits.

The subjective assessment revealed that most acne and rosacea patients recognized an impact of diet on their skin condition, emphasizing the importance of addressing nutrition in any treatment plan [29,30]. Overall, triggers were more clearly identified than beneficial foods, as evidenced by the number of responses. Reported acne triggers were consistent with existing findings in the literature, suggesting that the overall consumption of chocolate, fried foods, refined sugar, milk, alcohol, and dairy products should be individually limited [8,31,32]. Alcohol, specifically wine, was the leading self-reported rosacea trigger, reinforcing previous data showing that alcohol consumption is a known risk factor for rosacea [33]. Interestingly, several seemingly unrelated foods, including fruits, spices, chocolate, and citrus fruits, were also reported as rosacea triggers. A possible pathogenic mechanism underlying the exacerbation potential of these foods may be explained by the presence of cinnamaldehyde, a compound that triggers transient receptor potential (TRP) ion channels found on sensory nerves and keratinocytes. The activation of these channels causes the release of substance P, resulting in an inflammatory response and dilation of arterioles in rosacea skin [12,34]. Coffee was also a self-reported rosacea trigger. However, recent studies have shown that coffee drinkers actually have a lower likelihood of developing rosacea, possibly due to the vasoconstrictive effects of caffeine [35,36,37]. It should be considered that the triggering effect in the present cohort may have been influenced by the vasodilatory effects of hot beverages in general [38].

The food items reported to be beneficial for both the AG and RG can be classified as a Mediterranean diet (MD), characterized by a high consumption of vegetables, legumes, oily fish, olive oil, nuts, and only moderate intake of meat, cheese, and alcohol [39,40,41]. While the MD is generally known to be beneficial for health and conditions such as diabetes, cancer, and cardiovascular disease [42,43], the data from this study suggest additional advantageous effects on facial inflammatory dermatoses. This supports recent findings of a negative correlation between acne and the adherence to a MD [44], as well as a reduced incidence of rosacea with adherence to a MD [39]. Thus, adhering to a MD appears to be beneficial for both acne and rosacea. Omega-3 fatty acids, available in oily fish, algae, nuts, and seeds, along with probiotics found in fermented vegetables, are currently being studied as potential food items to reduce inflammatory skin conditions. Several mechanisms have been proposed to elucidate their potentials, with acne exhibiting more data than rosacea [45,46,47]. Omega-3 fatty may modulate sebum production, reduce inflammatory cytokines, inhibit Cutibacterium acnes growth, enhance skin barrier function, and provide antioxidant properties [5,45,48,49]. Oral probiotics could restore an imbalanced gut microbiome, leading to favorable effects on distant sites, including the epidermal barrier function of the skin [50].

Interestingly, the analysis of patients’ actual dietary intake based on significant results in the FFS contradicted the self-reported beneficial and aggravating foods. For example, although perceived as beneficial, acne patients consumed significantly fewer fruits and vegetables than the control group. Remarkably, acne patients reported a lower consumption of milk compared to the ACG, suggesting that they were aware of milk and dairy products as dietary acne triggers and had adjusted their eating habits accordingly. Milk and dairy products can increase insulin and IGF-1 levels and activate the nutrient-sensitive kinase mammalian target of rapamycin complex-1 (mTORC1) [51], which promotes anabolic pathways associated with increased seborrhea and follicular hyperkeratosis, both involved in acne development [52,53]. Laboratory analysis revealed associations between diet and these biomarkers, particularly in the AG. Significantly elevated IGF-1 levels were found in the AG compared to the ACG and RG. More frequent dairy intake in acne patients was associated with elevated IGF-1 levels compared to patients with normal IGF-1 levels. These findings suggest that diet may directly influence biological factors involved in the pathogenesis of these diseases. Future studies should investigate whether IGF-1 levels could be used as a screening tool to assess dairy intake in acne patients.

Patients with rosacea consumed significantly more animal products compared to controls, despite being self-reported as dietary triggers, and fewer legumes, despite being perceived as beneficial. Red and processed meat intake may have contributed to increased LDL, total cholesterol, and triglycerides, as seen in the laboratory analysis of the RG compared to the RCG and AG [54]. Fried foods, processed meats such as ham and burgers, and aged cheeses are high in histamine, with isolated studies suggesting possible effects on rosacea skin [12]. However, future research is needed to evaluate possible associations between meat intake and rosacea, as data are currently lacking. Similarly, the effects of soy on both acne and rosacea have not yet been investigated, with limited intake in the present acne and rosacea cohorts compared to individual control groups.

Dietary scores were derived from patient FFS responses. As seen in the subjective analysis of dietary beneficial and triggering food items, acne patients’ responses were more consistent, resulting in a more accurate score and higher *p* value compared to rosacea. The acne score included 13 items and resulted in a calculated odds ratio of 14.5, while the rosacea score included 7 items with a calculated odds ratio of 5.5. According to the ANS, daily intake of water, fresh fruit, and coffee; weekly intake of vegetables, unsweetened tea, bread, cheese, and pasta; monthly intake of oats, dairy products, cooked fruit, wine; and limited intake of ham were significantly associated with a decreased risk of acne. While these food items not only reflect a MD, further supporting that this dietary style may reduce the risk of acne, they also include the most frequent self-reported beneficial acne food items from the subjective assessment.

According to the RNS, regular coffee and nut consumption of more than three times per week was associated with a decreased risk of rosacea. Interestingly, the proposed score supports the suggestion in the previous literature that coffee does not increase the risk of rosacea and highlights possible benefits of nuts [36]. As nuts, such as walnuts, are a valuable source of omega-3 fatty acids with anti-inflammatory properties, they may play a protective role, although further studies are needed to investigate the exact association with rosacea. Strictly limiting the intake of animal products, including processed meats and fried foods, has been shown to be associated with a reduced risk of rosacea. These findings have not been reported in the literature and, interestingly, are partially consistent with the subjective trigger assessment of the present cohort. Further validation is needed by evaluating the results in larger patient populations.

The use of dietary supplements was reported more frequently in the RG than in the AG. Although patients may take oral supplements with the intention of improving their health, there are currently no clinical recommendations suggesting that supplementation may be beneficial for acne and rosacea in the absence of existing deficiencies. As studies have even reported cases of acne and rosacea triggered by oral supplementation, including B 12 vitamin, clinicians should always critically evaluate their use alongside patients’ daily medications [55,56].

The impact of acne on patients’ quality of life was found to be more pronounced compared to rosacea, as indicated by the mean DLQI scores. However, no significant difference in the EQ-5D-EL scores was observed between the AG and RG. Both the AG and RG reported a significant decrease in quality of life and overall health compared to their respective control groups, and stress was a trigger for both acne and rosacea, consistent with the recent literature [57].

To the best of our knowledge, the present study proposes a new way to approach the challenging topic of diet and its impact on disease risk. By proposing the first disease-specific risk scores for acne and rosacea attributable to patients’ dietary habits, the aim was to help clinicians to concretize clinical recommendations and provide clearer information for patients. Although further interventional studies are needed, assessing patients’ dietary habits in the presented manner may empower them to make informed lifestyle choices and promote long-term adherence to positive dietary changes.

It is important to note that this study has several limitations. Because dietary habits vary widely between cultures and countries, the results of the present German cohort may not be generalizable to a worldwide population. In addition, self-reported dietary habits always carry the risk of recall bias, and the cross-sectional design has shortcomings compared to placebo-controlled, interventional, randomized trials. Further research with larger sample sizes is warranted to validate and extend these findings.

## 5. Conclusions

The results emphasize that dietary factors have an impact on acne and rosacea. Triggers were more clearly identified by subjective assessment than beneficial foods. Dietary scores were established that may serve as useful tools for assessing acne and rosacea risk based on dietary patterns. Clinical recommendations can be made more clearly for acne than for rosacea, but this study serves as a first step for concretizing dietary suggestions for both groups.

## Figures and Tables

**Table 1:** Demographic characteristics.

	AG	ACG	RG	RCG
**Participants**	n = 120	n = 32	n = 105	n = 39
**Gender, n (%)**				
Female	76 (63)	22 (69)	75 (71)	31 (80)
Male	44 (37)	10 (31)	30 (29)	8 (20)
**Mean age, y (±SD)**	23.3 (±7.1) *^†^*	25.5 (±7.7)	46.1 (±15)	40.9 (±11.6)
**Range of age, y**	43	41	61	40
**BMI, kg/m^2^**	23.1 *^†^*	22.9	24.8	23.4
**Body fat, %**	**25.3** ^†^*^†^*	30.1	36	34.2
**Clinical acne assessment, n (%)**				
Acne comedonica	29 (24)			
Acne papulopustulosa	84 (70)			
Acne conglobata	7 (6)			
**Clinical rosacea assessment, n (%)**				
Persistant erythema/telangiectasia			65 (62)	
Papules/pustules			34 (32)	
Phymata			5 (5)	
Ocular symptoms			1 (1)	
**Mean disease persistence, y**	5.9		6.6	
**Regular supplement intake, n (%)**	52 (43.3) * **^#^**	19 (59.4)	62 (59.6)	26 (66.7)
**Facial sebum, mean (µg/cm^2^)**	**136.3 ^†^**	89	136.7 ^†^	103.2
**DLQI, mean (±SD)**	**6.5 ^†^** ***^†^* (±5.0)**	1.6 (±3.4)	**5.4 ^†^ (±5.6)**	0.67 (±1.3)
**EQ-5D-3L, mean (±SD)**	**76.5 ^†^ (±18.2)**	89.7(±11.8)	**73.04 ^†^ (±19.5)**	89.15 (±8.4)

(AG = acne group, ACG = acne control group, RG = rosacea group, RCG = rosacea control group, BMI = body mass index, DLQI = Dermatology Life Quality Index, EG-5D-3L = European Quality of Life 5 Dimension 3 Level, n = number, SD = standard deviation, y = year); * = *p* < 0.05 AG vs. ACG, RG vs. RCG; **^#^** = *p* < 0.05 AG vs. RG; ^†^ = *p* < 0.01 AG vs. ACG, RG vs. RCG; *^†^* = *p* < 0.01 AG vs. RG.

**Table 2:** Significant results of the standardized food frequencies survey (FFS) comparing acne patients (AG) and the acne control group (ACG).

Food Items	AG	ACG	*p*-Value
Water, mean (±SD)	8.57 (±1.88)	9.19 (±1.87)	0.004
Coffee, mean (±SD)	3.63 (±3.12)	5.13 (±3.05)	0.010
Wine, mean (±SD)	0.68 (±0.99)	1.16 (±1.19)	0.024
Fruits, mean (±SD)	0.34 (±1.08)	0.94 (±1.13)	0.000
Vegetables, mean (±SD)	2.88 (± 2.11)	4.03 (±2.31)	0.015
Bread, mean (±SD)	3.29 (± 2.30)	2.06 (±1.56)	0.009
Pasta, mean (±SD)	2.98 (±1.46)	3.34 (±0.79)	0.048
Boiled potatoes, mean (±SD)	2.20 (±1.39)	2.63 (±1.56)	0.006
Cows‘ milk, mean (±SD)	3.81 (±2.69)	4.87 (±2.84)	0.047
Soy products, mean (±SD)	0.78 (±1.12)	1.41 (±1.29)	0.001
Coconut oil, mean (±SD)	0.18 (±0.39)	0.44 (±0.50)	0.003

(SD = standard deviation).

**Table 3:** Significant results of the standardized food frequencies survey (FFS) comparing rosacea patients (RG) and the rosacea control group (RCG).

Food Items	RG	RCG	*p*-Value
Soy products, mean (±SD)	0.77 (±1.103)	1.38 (±1.227)	0.001
Honey/Jam, mean (±SD)	2.76 (±2.096)	1.64 (±1.597)	0.004
Ham, mean (±SD)	1.73 (±1.595)	0.9 (±1.071)	0.006
Burger/Kebab, mean (±SD)	0.7 (±0.887)	0.38 (±0.847)	0.015
Meat, mean (±SD)	2.22 (±1.387)	1.54 (±1.411)	0.016
Fried potatoes, mean (±SD)	1.3 (±1.218)	0.82 (±1.121)	0.023
Coffee, mean (±SD)	5.19 (±2.839)	6.46 (±1.998)	0.027
Legumes, mean (±SD)	1.78 (±1.38)	2.54 (±1.89)	0.041

(SD = standard deviation).

**Table 4:** (**a**): Cut-off consumption frequencies and corresponding odds ratios. Frequencies above the respective cut-off result in increasing odds ratios for acne. (**b**): Frequencies below the cut-off result in increasing odds ratios for acne.

(**a**)			
**Food Items**	**Cut-Off Frequency**	**OR**	***p*** **-Value**
Ham	≥2/wk	3.37	0.026
Cornflakes	≥1/mth	2.14	0.085
Pork/Beef	≥4/wk	1.93	0.178
Bread	≥1/d	*^#^*	0.002
(**b**)			
**Food Items**	**Cut-Off Frequency**	**OR**	***p*** **-Value**
Pasta	≤2/mth	6.95	0.004
Rice	≤2/wk	6.2	0.079
Cooked/Stewed Fruits	never	5.35	0.000
Dairy products	≤1/mth	5.22	0.001
Water	≤4/d	3.42	0.003
Vegetables	≤3/wk	3.35	0.003
Oats	≤1/mth	2.85	0.016
Unsweetened tea	≤3/wk	2.64	0.016
Coffee	≤5/wk	2.63	0.018
Fresh fruit	≤1/d	2.42	0.049
Ice cream	never	2.16	0.071
Cheese	≤1/wk	2.27	0.040
Wine	never	2.27	0.040

(OR = odds ratio, d = day, mth = month, wk = week, ^#^ = no calculated odds ratio resulting from missing comparable statement in control group).

**Table 5:** (**a**): Cut-off consumption frequencies and corresponding odds ratios. Frequencies above the respective cut-off result in increasing odds ratios for rosacea. (**b**): Frequencies below the cut-off result in increasing odds ratios for rosacea.

(**a**)			
**Food Items**	**Cut-Off Frequency**	**OR**	***p*** **-Value**
Honey/Jam	≥2/mth	2.82	0.006
Ham	≥2/mth	3.44	0.002
Meat	≥2/mth	3.208	0.002
Fried potatoes	≥2/mth	1.99	0.089
Burger/Kebab	≥1/mth	2.92	0.01
(**b**)			
**Food Items**	**Cut-Off Frequency**	**OR**	***p*** **-Value**
Coffee	≤2/wk	6.25	0.007
Nuts	≤2/wk	2.37	0.023

(OR = odds ratio, d= day, mth = month, wk = week).

**Table 6:** (**a**): Proposition of the Acne Nutrition Score (ANS). Higher score results in a decreased risk for acne. (**b**): Distribution of scores among AG and ACG.

(**a**)					
**ANS—QUESTIONS**			**CRITERIA**	**YES**	**NO**
1. Do you drink **water**.			≥5x/day		
2. Do you eat **fresh fruit**.			≥2x/day		
3. Do you drink **coffee**.			≥1x/day		
4. Do you eat **vegetables**.			≥5x/week		
5. Do you drink **unsweetened tea.**			≥5x/week		
6. Do you eat **cheese**.			≥3x/week		
7. Do you eat **bread**.			≥1x/week		
8. Do you eat whole-grain **pasta**.			≥1x/week		
9. Do you eat **oats**.			≥3x/month		
10. Do you eat **dairy products.**			≥3x/month		
11. Do you eat **cooked fruits.**			≥1x/month		
12. Do you drink **wine**.			≥1x/month		
13. Do you eat **ham**.			≤3x/month		
**TOTAL SCORE (total number of “yes” answers)**					
(**b**)					
**Score**	**AG, n (%)**	**ACG, n (%)**	**OR**	***p*** **-VALUE**	
≤7 points	102 (91.9)	9 (8.1)	14.48	<0.001	
≥8 points	18 (43.9)	23 (56.1)			

(n = number, green = daily frequencies, blue = weekly frequencies, red = monthly frequencies); ≤7 points → Increased risk; ≥8 points → Decreased risk.

**Table 7:** (**a**): Proposition of the Rosacea Nutrition Score (RNS). Higher score results in a decreased risk for rosacea. (**b**): Distribution of scores among RG and RCG.

(**a**)					
**RNS—QUESTIONS**			**CRITERIA**	**YES**	**NO**
1. Do you drink **coffee**.			≥3/week		
2. Do you eat **nuts**.			≥3/week		
3. Do you eat **honey/jam**.			≤1x/month		
4. Do you eat **ham**.			≤1x/month		
5. Do you eat **meat**.			≤1x/month		
6. Do you eat **fried potatoes.**			≤1x/month		
7. Do you eat **burger/kebab.**			never		
**TOTAL SCORE (total number of “yes” answers)**					
(**b**)					
**Score**	**RG, n (%)**	**RCG, n (%)**	**OR**	***p*** **-VALUE**	
≤4 points	77 (85.6)	13 (14.4)	5.5	<0.001	
≥5 points	28 (51.90)	39 (27.1)			

(n = number, blue = weekly frequencies, red = monthly frequencies, yellow = abstinence); ≤ 4 points → Increased risk; ≥ 5 points → Decreased risk.

**Table 8:** Laboratory parameters of the study population.

	AG		ACG		RG		RCG	
**IGF-1**, ng/mL, mean (±SD), n	**297.55 ^†^** ***^†^***	117	244.63	32	158.30	102	166.97	38
	(±104.30)		(±111.82)		(±56.88)		(±58.96)	
**Glucose**, mg/dL, mean (±SD), n	**82.70** ***^†^***	117	84.41	32	87.06	102	86.79	38
	(±9.78)		(±12.29)		(±10.31)		(±20.13)	
**HOMA Index**, mean (±SD), n	4.00	107	5.26	32	**2.93 ^†^**	101	2.64	38
	(±6.40)		(±9.80)		(±2.68)		(±5.09)	
**HbA1c**, mmol/mol, mean (±SD), n	**33.38** ***^†^***	117	33.03	32	34.87	102	34.47	38
	(±3.40)		(±3.17)		(±4.69)		(±5.00)	
**Fructosamine**, µmol/L, mean (±SD), n	265.72	117	266.28	32	267.13	102	272.26	38
	(±21.44)		(±21.59)		(±20.63)		(±23.65)	
**Cholesterol**, mg/dL, mean (±SD), n	**167.26** ***^†^***	117	177.75	32	231.74	102	197.13	38
	(±30.99)		(±38.37)		(±22.77)		(±36.93)	
**LDL**, mg/dL, mean (±SD), n	**92.93** ***^†^***	116	100.44	32	126.26	101	115.32	38
	(±26.86)		(±35.48)		(±38.15)		(±34.62)	
**TG**, mg/dL, mean (±SD), n	**91.01 ^†^** ***^†^***	117	128.94	32	**129.93 ^†^**	102	86.45	38
	(±51.72)		(±118.71)		(±79.32)		(±40.01)	
**CRP**, mg/dL, mean (±SD), n	0.19	117	0.24	32	0.26	102	0.24	38
	(±0.22)		(±0.35)		(±0.19)		(±0.24)	
**Leukocytes**, G/L, mean (±SD), n	7.27	117	6.78	32	**6.68 ^†^**	102	5.72	38
	(±1.78)		(±1.76)		(±1.80)		(±1.78)	
**Vitamin D**, ng/mL, mean (±SD), n	22.71	117	25.05	32	27.24	102	23.92	38
	(±12.12)		(±23.41)		(±23.88)		(±10.83)	
**Zinc**, µg/dL, mean (±SD), n	82.91	115	83.26	31	85.11 * ***^#^***	102	90.68	38
	(±16.53)		(±12.08)		(±17.12)		(±14.81)	

(AG = acne group, ACG = acne control group, RG = rosacea group, RCG = rosacea control group. CRP = C-reactive protein, dL = deciliter, G = giga, HOMA*-I*ndex = Homeostasis Model Assessment = (Insulin × Glucose)/405, IGF-1= insulin-like growth factor 1, LDL = low-density lipoprotein-cholesterol, L = liter, mg = milligram, ng = nanogram, n = number, SD = standard deviation, TG = triglycerides). * = *p* < 0.05 AG vs. ACG, RG vs. RCG; **^#^** = *p* < 0.05 AG vs. RG; ^†^ = *p* < 0.01 AG vs. ACG, RG vs. RCG;*^†^*= *p* < 0.01 AG vs. RG.

## Data Availability

The data presented in this study are available on request from the corresponding author.
